# Concurrent muscle and bone deterioration in a murine model of cancer cachexia

**DOI:** 10.1002/phy2.144

**Published:** 2013-11-07

**Authors:** EunHi Choi, Kadir Carruthers, Li Zhang, Nathan Thomas, Ricardo A Battaglino, Leslie R Morse, Jeffrey J Widrick

**Affiliations:** 1 Department of Physical Medicine and Rehabilitation, Spaulding Rehabilitation Hospital and Harvard Medical SchoolBoston, Massachusetts; 2 Deparment of Physical Medicine and Rehabilitation, Hallym University College of MedicineGangwon-do, South Korea; 3 Forsyth InstituteCambridge, Massachusetts

**Keywords:** Body composition, bone mineral density, muscle function, muscle strength

## Abstract

Cachexia is defined as an excessive, involuntary loss of fat and lean tissue. We tested the validity of the Lewis lung carcinoma (LLC) as a model of cancer cachexia and examined its effect on the two major lean tissue components, skeletal muscle and bone. LLC cells (0.75 × 10^6^) were injected into the left thigh of C57BL/6 mice. Control mice received an equal volume injection of growth media. Tumors were observed in all LLC-injected animals 21 and 25 days post inoculation. LLC-injected animals showed significant reductions in fat and lean mass despite having the same average daily caloric intake as media-treated mice. Global bone mineral density (BMD) had fallen by 5% and 6% in the LLC animals at 21 and 25 days, respectively, compared to a BMD increase of 5% in the 25-day media-treated animals. Extensor digitorum longus (EDL) muscles (isolated from the noninjected hindlimb) showed earlier and quantitatively greater losses in mass, physiological cross-sectional area (pCSA), and tetanic force compared to soleus muscles from the same hindlimb. By the 25th day post-LLC inoculation, EDL force/pCSA was reduced by 19% versus media treatment. This loss in specific force was not trivial as it accounted for about one-third of the reduction in EDL absolute force at this time point. Muscle strips dissected from the diaphragm of LLC mice also exhibited significant reductions in force/pCSA at day 25. We conclude that LLC is a valid model of cachexia that induces rapid losses in global BMD and in limb and respiratory muscle function.

## Introduction

Several forms of cancer, and particularly those of the lung and viscera, are associated with a profound loss of fat and lean tissue, a condition known as cachexia. Skeletal muscle, which represents the largest lean tissue component of the body, may be reduced to 25% of its normal mass in severe cachexia while nonmuscle protein is relatively spared (Fearon 1992). Cachexia is present to some degree in approximately 50% of cancer deaths (Inagaki et al. 1974). Of these fatalities, about half can be directly attributed to the immobility and respiratory muscle insufficiency that result from extensive skeletal muscle wasting (Inagaki et al. 1974; Fearon 1992).

Bone, the second largest lean tissue compartment, also appears to be impacted by cachexia. Lung cancer patients who had lost 30% of their body mass showed significantly lower mineral content than their healthy-matched peers (Fearon 1992). However, bone loss in cachexia has been studied much less than muscle wasting despite the fact that cachexia-induced deterioration of the skeletal system could have critical long-term health consequences for cancer survivors. Kandarian (2008) has proposed that muscle and bone mass may be regulated in tandem during cachexia because a number of the signaling pathways that induce muscle wasting are also known to promote bone loss. Thus, interactions between muscle and bone may represent a scientifically and clinically important, but relatively unexplored, aspect of cachexia.

A better understanding of how cachexia impacts the musculoskeletal system requires the development and characterization of preclinical animal models for use in mechanistic studies. A number of tumor models have been used to induce skeletal muscle loss in laboratory rodents. However, many of these models have not been rigorously evaluated as models of cachexia. Nor is it clear whether they impact the skeletal system. Finally, various approaches used to quantify the effects of cachexia have produced somewhat contradictory findings that warrant further investigation.

As an example of the later, a number of functional studies have concluded that during cachexia, muscle force declines in direct proportion to losses in muscle mass or fiber size (Gorselink et al. 2006; Aulino et al. 2010; Murphy et al. 2011). This suggests that during cachexia, muscle cells show a reduction in their cross-sectional area but are otherwise normal. In contrast, molecular and histological approaches have revealed intracellular perturbations to muscles from cachexic animals that would be expected to detrimentally affect force measurements at tendons. These include disproportionate losses in the motor protein myosin and in the force-transmitting protein dystrophin (Acharyya et al. 2004, 2005) and an extensive disarrangement of the myofilament lattice (Aulino et al. 2010). In addition, many of the molecules that signal muscle wasting in cachexia, such as nuclear factor of kappa B (Wyke et al. 2004), TNF-*α* (Llovera et al. 1998a), and reactive oxygen intermediates (Buck and Chojkier 1996), have acute and/or persistent detrimental effects on cellular mechanisms of muscle contraction (Andrade et al. 1998; Cai et al. 2004; Hardin et al. 2008; Prochniewicz et al. 2008).

To address these issues, we evaluated caloric intake, global lean and fat mass, whole-body bone mineral density (BMD), and the functional properties of skeletal muscles of tumor-bearing mice. Tumors were induced using the Lewis lung carcinoma (LLC), a well-established tumor model (Mayo 1972). Injection of LLC cells into a hindlimb muscle has been used previously in studies of body composition, muscle atrophy, muscle protein turnover, and muscle function (Llovera et al. 1998b; Busquets et al. 2004; Nicholson et al. 2006; Argiles et al. 2008; Murphy et al. 2011). In preliminary work, we found intramuscular injection of LLC cells to be a more reliable and robust method of inducing tumor growth compared to subcutaneous injection (Azouz et al. 2008). We therefore injected LLC cells into one hindlimb of C57BL6 mice and several weeks later examined, (1) changes in body composition and BMD using small-animal dual energy X-ray absorptiometry (DXA), (2) ex vivo functional properties of representative oxidative and glycolytic limb muscles from the contralateral noninjected hindlimb, and (3) function of the critically important diaphragm (DIA). To identify any temporal effects of tumor burden, all variables were assessed after tumors were well established and again after several days of additional tumor growth.

## Methods

### Animal care procedures

All procedures involving animals were approved by the IACUC at the Forsyth Institute, Cambridge, MA. Male C57BL6 mice were obtained from Charles River Laboratories. All mice were 56 days of age upon arrival. The animal facility where the animals were housed was maintained at 23°C with a 12:12-h light-dark cycle. Throughout the study, mice had ad libitum access to tap water and a standard rodent diet (5053 PicoLab rodent diet 20, LabDiet, St. Louis, MO). Chow mass was determined at the start and conclusion of each study and an average caloric intake for each animal calculated by dividing this value by the number of days in the experiment.

### Lewis lung carcinoma

Lewis lung carcinoma cells (# CRL-1642) were purchased from American Type Culture Collection, Manassas, VA. Cells were cultured in Dulbecco's Modified Eagle's Medium (DMEM) containing 10% fetal bovine serum, 1% of 100 U/mL penicillin, and 100 *μ*g/mL streptomycin and were passaged every 3–4 days. For injections, cells were diluted in DMEM at a concentration of 3 × 10^6^ cells/mL.

### Experimental design

Mice were assigned to a media treatment group or an LLC treatment group. Assignment to media and LLC treatments was balanced in order to minimize group differences in initial body mass. When they were between 61 and 66 days old, mice were anesthetized with a xylazine/ketamine cocktail (0.1 mg xylazine/kg; 0.02 mg ketamine/kg) and the dorsal surface of the left limb shaved. A 250-*μ*l bolus of growth media containing 7.5 × 10^5^ LLC cells was injected into the left thigh of mice making up the LLC-treated group. Animals in the media-treated group received a similar injection containing growth media only. After determination of body composition (see below), the animals were returned to their individual cages and periodically monitored as they recovered from the anesthesia. Animals were studied again either 21 or 25 days post inoculation. These time points were chosen so that we could, (1) compare our results to previous functional studies that have studied tumor-bearing animals out to 21–22 days (Gorselink et al. 2006; Aulino et al. 2010), and (2) provide several days of additional tumor growth (Mayo 1972) in order to observe time-related effects of tumor burden.

### Dual energy X-ray absorptiometry

On the day of study, mice were lightly anesthetized with an intraperitoneal injection of sodium pentobarbital. Body composition and global (whole body minus the head) BMD were determined in vivo using a Lunar PIXImus small-animal DXA scanner (Lunar PIXImus2, software version 1.4X, GE Medical Systems Inc., Waukesha, WI). Lean mass was determined by subtracting fat mass from total tissue mass. To assess the contribution of the tumor to body composition, scans were repeated under the following conditions and the results were compared: (1) excluding the tumor from the scan area and (2) excising the tumor after sacrifice and rescanning the animal. To exclude the tumor from the scan an oval-shaped exclusion area was placed over the visible edges of the tumor. Using anatomical landmarks, a similar region was excluded in the baseline scans.

### Muscle function

After the posttreatment DXA measurements, animals were administered additional pentobarbital (i.p.) until they reached a surgical plane. The extensor digitorum longus (EDL) and soleus (SOL) from the right limb were then excised. SOL and EDL muscles were obtained from the right hindlimb in order to avoid any physical effect that the tumor mass may have had on the muscles of the left hindlimb. After limb muscle dissection, the DIA was excised. We focused only on these particular muscles because they have the following qualities: (1) taken together, they provide a wide range in fiber-type composition and metabolic profile, (2) they all have a relatively small radius for oxygen diffusion, which allowed us to study them ex vivo, and (3) they cover a range of physiological functions in that they represent a hindlimb extensor, a hindlimb flexor, and a respiratory muscle.

Muscles were pinned out at a slightly stretched length on a Sylgaar base in a small dish containing a modified bicarbonate buffer (composition, in mmol/L: 137 NaCl, 11 glucose, 5 KCl, 1.25 CaCl_2_, 1 MgSO_4_, 1 NaH_2_PO_4_, 24 NaHCO_3_, 0.025 tubocurarine chloride). The buffer was equilibrated with 95% O_2_ and 5% CO_2_. Small muscle strips, which included the central tendon and rib, were dissected from the costal DIA. Silk suture was used to suspended intact SOL and EDL muscles and DIA strips vertically in a tissue bath between a hook and an isometric force transducer (model 60-2996, Harvard Apparatus, Holliston, MA) as described previously (Widrick et al. 2011). The hook could be translated by a micrometer allowing fine changes in muscle length. The bath contained bicarbonate buffer that was continuously aerated with 95% O_2_ and 5% CO_2_ and maintained at 35°C.

Muscles were stimulated using 200-*μ*sec square wave pulses delivered to platinum electrodes flanking the muscle. A personal computer, data acquisition board (model 6229; National Instruments, Austin, TX), and custom program written in LabView (National Instruments) were used to control the output of a biphasic constant current muscle stimulator (model 701; Aurora Scientific Inc., Aurora, ON, Canada) while simultaneously recording muscle force to disk (1000 Hz). Stimulation current and muscle length were adjusted to maximize tetanic force during a 300 Hz, 150 msec stimulus train for EDL muscles, a 200 Hz, 500 msec train for SOL muscles, and a 200 Hz, 300 msec train for DIA strips. The length of the muscle giving maximum force was measured with digital calipers and recorded as optimal length (*L*_o_). To minimize fatigue, at least 3 minutes separated all contractions. Muscle physiological cross-sectional area (pCSA) was calculated by dividing muscle mass by the product of fiber length and muscle density. Fiber length was calculated using the fiber length to *L*_o_ ratios (0.71 for SOL, 0.44 for EDL, 1.00 for DIA) available in the literature (Brooks and Faulkner 1988; Stevens and Faulkner 2000). Muscle density was taken as 1.06 g/cm^3^ (Mendez and Keys 1960). Specific force, or stress, was defined as peak tetanic force divided by pCSA. Muscle force and stress were expressed in SI units (newtons and pascals, respectively).

### Statistical analysis

Data are presented as mean ± SE. For muscle variables, the four treatment conditions were analyzed by one-way analyses of variance (ANOVA). In cases where pre- and posttreatment data were obtained (body composition, bone), we conducted a repeated measures ANOVA (treatment × time) on the raw data with a follow-up one-way ANOVA on the change in each variable (postvalue minus prevalue) over time. In the case of significant main effects (ANOVA) or treatment × time interactions (repeated measures ANOVA), differences between means were identified using the Bonferroni post hoc procedure. All statistical analysis were performed using GraphPad Prism 6 (GraphPad Software Inc., La Jolla, CA) at an alpha error rate of *P* < 0.05.

## Results

### Tumor mass

All LLC-injected mice developed a semisolid tumor in the upper left hindlimb. Tumor masses reached an average of 3.9 ± 0.4 g (*N* = 13) at the 21-day time point and 5.7 ± 0.3 g (*N* = 15) after 25 days.

### Tumor-free body mass and caloric intake

**Figure 1:**
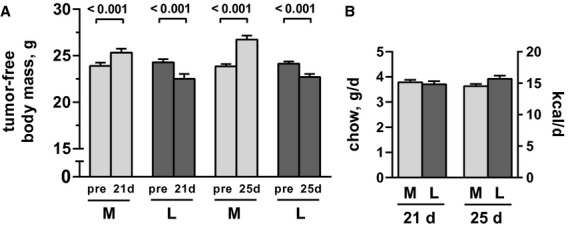
Body mass and caloric intake of media- (M) and Lewis lung carcinoma-(L) treated mice. (A) Pre- and posttreatment tumor-free body mass of 21 days treated (*N* = 13 M mice, 13 L mice) and 25 days treated (*N* = 15 M mice, 15 L mice) mice. ANOVA revealed a significant treatment × time interaction (*P* < 0.001). Differences between pre- and postmeans were evaluated by the Bonferroni post hoc procedure with horizontal lines connecting means that are significantly different (*P*-values above lines). (B) Daily chow consumption and corresponding caloric intake for the mice in (A). There were no differences in chow consumption or caloric intake between groups (*P* = 0.262).

Tumor-free body mass increased for the media-treated animals but declined for the LLC-treated animals (Fig. 1A). The 21-day and the 25-day LLC-treated groups showed similar changes in body mass (−1.8 ± 0.4 and −1.4 ± 0.2 g, respectively, *P* > 0.999). The opposing changes in the tumor-free mass of the media- versus LLC-treated mice occurred even though average daily chow consumption and caloric intake was similar between groups (Fig. 1B).

### Body composition

**Figure 2:**
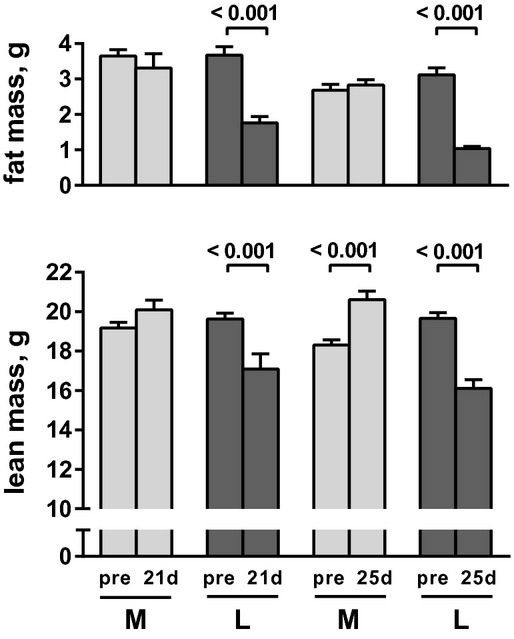
Body composition of media- (M) and Lewis lung carcinoma-(L) treated mice. Repeated measures ANOVA revealed a significant interaction between treatment and time for lean (*P* < 0.001) and fat (*P* < 0.001) masses. Differences between pre- and postmeans were evaluated by the Bonferroni post hoc procedure with horizontal lines connecting means that are significantly different (*P*-values above lines). *N* = 5 M and 10 L mice at 21 days; 11 M and 9 L mice at 25 days.

No change in lean mass was detected when determining body composition with the tumor present and included in the scan (data not shown). This is due to the composition of the tumor itself which is composed of lean mass, thereby obscuring loss of total body lean mass. When the tumor was excluded from the scan, lean and fat mass were unchanged in 21-day media-treated mice, but declined by 2.5 and 1.9 g, respectively, in their LLC-treated counterparts (Fig. 2). At the 25-day time point, media-treated mice showed an increase in lean mass with no change in fat mass, whereas lean and fat mass fell 3.6 and 2.1 g, respectively, in the LLC-treated animals. There were no statistically significantly differences between LLC 21-day versus LLC 25-day losses in either fat mass (*P* > 0.999) or lean mass (*P* = 0.446), that is, an additional 4 days of tumor growth did not effect changes in the body composition in the LLC animals. Similar results were obtained from a subset of animals in which the tumor was excised after sacrifice but prior to the DXA scan (data not shown).

### Bone mineral density

**Figure 3:**
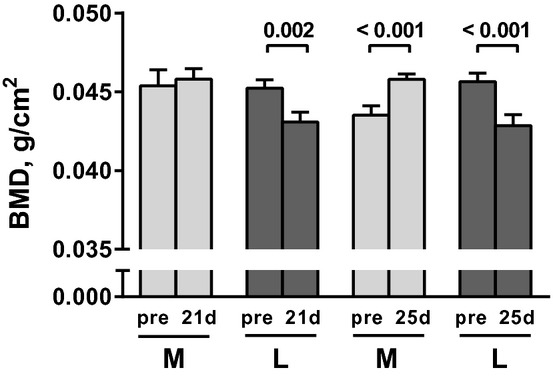
Bone mineral density (BMD) of media-treated (M) and Lewis lung carcinoma-treated (L) mice. Repeated measures ANOVA revealed a significant interaction between treatment and time (*P* < 0.001). Differences between means were evaluated by the Bonferroni post hoc procedure with horizontal lines connecting means that are significantly different (*P*-values above lines). Number of animals same as in Figure 2.

Bone mineral density was reduced by the LLC model. After 21 days of LLC treatment, BMD had fallen by 5% from the pretreatment value (Fig. 3). Extending LLC treatment an additional 4 days resulted in a 6% reduction in BMD, a change that was not statistically significantly different from the LLC 21-day response. In contrast, media-treated mice showed either no change (21 days), or an increase (25 days), in BMD over the course of the study.

### Limb muscle mass, pCSA, and tetanic force

**Figure 4:**
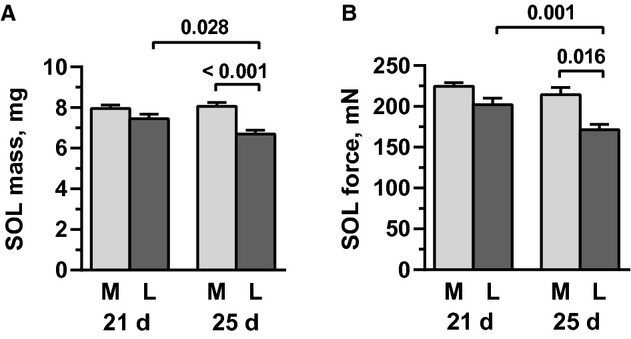
Properties of soleus (SOL) muscles from media-treated (M) and Lewis lung carcinoma-treated (L) mice. (A) Muscle mass. (B) Tetanic force. ANOVA revealed significant main effects for SOL mass (*P* < 0.001) and SOL force (*P* < 0.001). Differences between means were evaluated by the Bonferroni post hoc procedure with horizontal lines connecting means that are significantly different (*P*-values above lines). Number of muscles: 9 M and 11 L at 21 days; 9 M and 9 L at 25 days.

**Figure 5:**
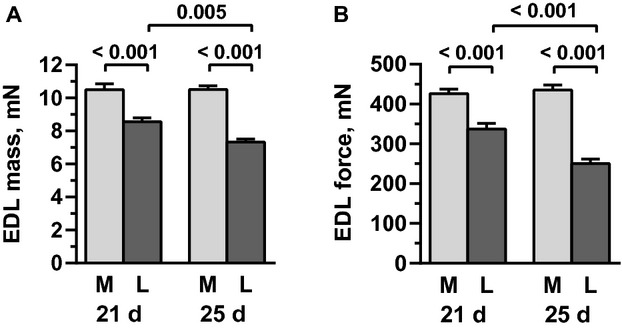
Properties of extensor digitorum longus (EDL) muscles from media-treated (M) and Lewis lung carcinoma-treated (L) mice. (A) Muscle mass. (B) Tetanic force. ANOVA revealed significant main effects for EDL mass (*P* < 0.001) and EDL force (P < 0.001). Differences between means were evaluated by the Bonferroni post hoc procedure with horizontal lines connecting means that are significantly different (*P*-values above lines). Number of muscles: 11 M and 12 L at 21 days; 9 M and 10 L at 25 days.

Twenty-one days of LLC treatment had no effect on SOL muscle mass, pCSA (0.854 ± 0.024 mm^2^ vs. 0.787 ± 0.020 mm^2^ for media- and LLC-treated, respectively), or tetanic force (Fig. 4). Four additional days of exposure to LLC reduced the mass, pCSA (0.843 ± 0.016 mm^2^ vs. 0.699 ± 0.019 mm^2^, *P* < 0.001), and tetanic force of this muscle by 17–20%. The EDL was more sensitive to LLC. By the 21-day time point, the EDL has already experienced a 18% loss in mass, a 14% reduction in pCSA (1.67 ± 0.05 mm^2^ vs. 1.43 ± 0.03 mm^2^, P < 0.001), and a 21% decline in tetanic force (Fig. 5). Unlike the changes in body mass, body composition, and BMD described above, 4 days of additional tumor growth resulted in further reductions in EDL mass, pCSA, and tetanic force (LLC 25-day values all significantly different from the corresponding LLC 21-day values). For example, EDL mass was reduced by 30%, physiological cross sectional by 29% (1.70 ± 0.03 mm^2^ vs. 1.21 ± 0.03 mm^2^, *P* < 0.001), and tetanic force by 43% in comparison to the 25-day media-treated values.

### Limb and respiratory muscle-specific force

**Figure 6:**
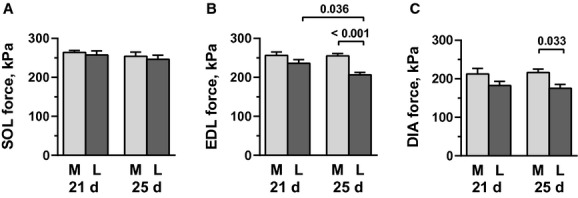
Specific force of soleus (SOL (A), extensor digitorum longus (EDL (B), and diaphragm (DIA (C), muscles from media-treated (M) and Lewis lung carcinoma-treated (L) mice. ANOVA revealed significant main effects for EDL (*P* < 0.001) and DIA (*P* = 0.022) muscles, but not for the SOL (*P* = 0.656). Differences between means were evaluated by the Bonferroni post hoc procedure with horizontal lines connecting means that are significantly different (*P*-values above lines). Number of SOL and EDL muscles same as in Figures 4 and 5, respectively. Number of DIA muscle preparations: 10 M and 12 L at 21 days; 11 M and 13 L at 25 days.

At 21 days post-LLC inoculation, specific force was similar to media-treated values for all three muscles studied (Fig. 6). However, extending exposure to LLC to 25 days resulted in a 19% reduction in the specific force of both the EDL and the DIA.

## Discussion

The LLC is a commonly used method of inducing tumor growth in laboratory mice. The cell line arose as a spontaneous carcinoma in the lung of a C57BL mouse in the early 1950s (see Mayo 1972). The LLC model is frequently used to study alterations in body composition, protein turnover, muscle mass, and muscle function in cachexia (Llovera et al. 1998b; Busquets et al. 2004; Nicholson et al. 2006; Argiles et al. 2008; Benny Klimek et al. 2010; Murphy et al. 2011).

The time course of LLC tumor growth and final tumor mass observed in this study are similar to earlier work using this model. Mayo (1972) implanted LLC tumor fragments subcutaneously in over 200 mice and reported average tumor masses of 4.6 and 6.4 g at days 20 and 24, respectively, with a median survival time of 25 days. The tumor masses we observed in this study, 3.9 and 5.7 g at 21 and 25 days, respectively, are somewhat less than those reported by Mayo and may reflect a delay in tumor formation when using an injected cell bolus versus the implantation of established tumor fragments. Consistent with this idea, we observed no mortality at 25 days posttumor injection.

To confirm that the LLC model is a valid model of cachexia, we quantified lean and fat compartments using small-animal DXA. Our results indicate that LLC treatment not only prevented the normal growth of the young mice studied here, but resulted in significant losses in their lean and fat tissue masses. These observed losses of lean and fat tissue could not be attributed to a reduced caloric intake by the LLC-treated animals. In fact, if chow consumption was calculated on a tumor-free body mass or lean mass basis, the caloric intake of the tumor-bearing mice would have exceeded that of their media-treated counterparts. Therefore, LLC-induced reductions in lean and fat tissue mass could not be attributed to reduced calorie intake, findings which validate the use of LLC as an experimental model of cachexia.

Bone loss was detectable at 21 days in the LLC-treated animals and became more pronounced at 25 days. This reduction in global BMD reached 5–6% in LLC animals, a difference that is even more noteworthy when one considers that in our growing animals, BMD increased by an average of 5% in the media-treated animals (day 25). We cannot exclude the possibility that an element of focal bone loss from the injected hindlimb side contributed to the total bone loss reported in this study. Alternative methods, such as microCT, may be required to identify small side-to-side hindlimb regional differences in bone density. However, it seems unlikely that focal bone loss near the injection site can entirely explain the 5–6% total body bone loss observed here.

The magnitude of this change in whole-body BMD approximates that observed for mice subjected to 21 days of hindlimb suspension (Spatz et al. 2013) or studied after 2 months of ovariectomy (Kim et al. 2013). This rapid and severe bone loss would be associated with increased fracture risk and is therefore clinically relevant. The mechanisms of bone loss in this model are unknown, but are not due to bony metastasis and may be multifactorial in nature. Previously, mineral content was reported to be significantly lower in cancer patients compared with matched controls (Fearon 1992), but to our knowledge, this is the first report of reduced BMD in an animal model of cancer cachexia. The present work indicates that the LLC mouse may be an appropriate model for use in preclinical studies designed to address this underresearched aspect of cancer cachexia.

We observed atrophy in all muscles studied, although the EDL was considerably more sensitive to wasting than the SOL. Not only did atrophy occur earlier in the EDL, but at the final time point studied, the EDL had experienced an almost twofold greater relative loss of mass and pCSA compared to the SOL. This greater sensitivity of the EDL to wasting is consistent with a previous LLC study (Busquets et al. 2004) and with other cancer models, such as the C-26 carcinoma (Diffee et al. 2002; Acharyya et al. 2004) and Apc^Min/+^ mice (Baltgalvis et al. 2008). In human cancer patients, fast muscle fibers have been reported to undergo more extensive atrophy than slow fibers (Mendell and Engel 1971). The present results support this finding as the C57BL/6 mouse SOL and EDL comprised ∼50 and 95% fast myosin, respectively (Agbulut et al. 2003).

Our data reveal that reductions in muscle mass alone are not the sole explanation for the force deficits that occur with cachexia. Most notably, both the EDL and the DIA showed ∼20% losses in force per unit pCSA (specific force) when tumor growth was extended to 25 days. A loss in EDL or DIA peak-specific force in tumor-bearing mice differs from the findings of previous studies. This is most likely because previous work, (1) studied mice with much lower tumor load than in this study (Murphy et al. 2011), or (2) made their final measurements 21–22 days after tumor inoculation (Gorselink et al. 2006; Aulino et al. 2010), a time at which we also observed no loss in specific force.

Because we used an ex vivo preparation, our findings can be attributed directly to the muscle tissue. In contrast, functional assays conducted in vivo or in situ can be impacted by several physiological processes that are absent or controlled in an ex vivo preparation. These include neuronal action potential propagation, action potential propagation across the neuromuscular junction, and blood flow. Although these other factors may be important physiological features of cachexia, focusing on the muscle tissue per se was a priority because our long-term goal is to understand how cellular mechanisms of contraction are altered in tumor-bearing animals. An ex vivo approach also enabled us to study the mechanical properties of the critically important DIA muscle using methodology identical to that used for the SOL and EDL.

It is important to note that the reduction in specific force reported here is of sufficient magnitude to be physiologically relevant. Because EDL absolute force dropped 43%, but pCSA only 29%, the loss in specific force accounts for ∼33% of the total force loss experienced by the muscle. While we cannot make similar calculations for the DIA (we are unable to calculate an absolute force deficit for this muscle because the size of the dissected muscle strip, and hence its absolute force, is an uncontrolled variable), the reduction in specific force of this muscle was similar to that of the EDL. A loss of DIA-specific force may be a critical factor contributing to respiratory failure in the later stages of cachexia. Conversely, reversing this loss of specific force may be an important intervention for maintaining respiratory function.

The reductions in specific force reported here suggest a change in cellular and molecular mechanisms responsible for force production. Because we studied limb muscles contralateral to the tumor site, as well as the DIA, these changes cannot be attributed to regional effects of the tumor, such as altered blood flow, but instead point to systemic mechanisms. Tumors secrete factors, or induce the host to produce factors, that increase protein degradation in skeletal muscle cells (for review, see (Tisdale 2009). It should be noted that protein degradation in cancer cachexia appears to be preferential, with the motor protein myosin particularly susceptible to loss (Acharyya et al. 2004; Banduseela et al. 2007). This preferential loss of myosin, along with gross disorganization of sarcomere ultrastructure (Banduseela et al. 2007; Aulino et al. 2010), would all be consistent with a loss of muscle-specific force.

In addition to direct loss of contractile protein, several of the molecules that signal elevated proteolysis in cancer cachexia, such as TNF-*α* (Llovera et al. 1998a) and NF-*κ*B (Cai et al. 2004), have been shown to have depressive effects on muscle contractility (Wilcox et al. 1994; Reid et al. 2002; Cai et al. 2004; Hardin et al. 2008). Cachexia is also associated with elevations in reactive oxygen intermediates (Buck and Chojkier 1996) and above normal levels of oxidants depress muscle contractility (Andrade et al. 1998; Prochniewicz et al. 2008). It is important to note that at least some of the effects of elevated TNF-*α* (Reid et al. 2002; Hardin et al. 2008) and reactive oxygen intermediates (Andrade et al. 1998; Prochniewicz et al. 2008) would be expected to be acute or qualitative, that is, an immediate, direct impact on cross-bridge function versus their potential longer term quantitative effects on cross-bridge number.

It is possible that the transmission of force to the tendons may be impaired in cachexia. Dystrophin, a cytoskeletal protein, has been reported to be reduced in tumor-bearing mice (Acharyya et al. 2005). Dystrophin is thought to protect the sarcolemma from damage during contraction and to assist in transmitting force from actin filaments to the extracellular matrix (Petrof et al. 1993; Rybakova et al. 2000). Therefore, a loss of this protein may contribute to reductions in the force produced by the muscle–tendon complex. Finally, the observed loss in specific force could arise from events occurring at the tissue level, that is, a shift toward a relatively greater amount of noncontractile material in the muscle.

Future studies will need to identify which of the above mechanisms is responsible for reductions in specific force. Expressing force relative to myofibrillar protein content (Taylor and Kandarian 1994), as suggested by one reviewer, may be useful in identifying whether changes in noncontractile components play a role in the loss in specific force but this approach cannot distinguish between normal cross-bridges and cross-bridges that have impaired function (Faulkner et al. 1999). Likewise, ultrastructural analyses (Aulino et al. 2010) can quantify a reduction in cross-bridge number but not the functional state of those cross-bridges that remain. These and other biochemical, molecular, and ultrastructural approaches will need to be combined with single fiber functional assays (Banduseela et al. 2007; Prochniewicz et al. 2008) in order to identify the mechanisms responsible for the specific force losses observed here. Regardless of the mechanisms involved, the present results demonstrate that functional deficits in some muscles of tumor-bearing mice exceed losses in the muscles' pCSA. Thus, changes in muscle mass are unlikely to be an appropriate surrogate measure of muscle function during cachexia, a finding that needs to be considered in the design of future experiments.

It should be noted that the tumor-bearing mice in the present work became subjectively less active as the study progressed. This raises the possibility that the bone loss and muscle functional deficits we observed were brought about by reductions in normal physical activity. In terms of muscle, this interpretation is opposed by our finding that the DIA of 25-day tumor-bearing mice also showed deficits in specific force. It therefore seems unlikely that specific force deficits can be explained entirely, if at all, by tumor-induced reductions in the normal physical activity of the animals. Future studies will need to measure and systemically manipulate physical activity of tumor-bearing animals in order to better understand potential interactions between physical activity, tumor growth, and bone and muscle physiology.

In summary, we have shown that the LLC model has no effect on the caloric intake of C57 mice, yet induces substantial reductions in both their lean and fat mass compartments. We observed substantial losses in BMD in tumor-bearing animals and significant muscle atrophy and force deficits in both limb and respiratory muscles. Muscles comprised predominately of fast fibers showed more severe forces losses as well as an additional loss in specific force, suggesting that cellular mechanisms of force production and/or transmission are impacted by cachexia. These deficits in BMD and muscle-specific force were not physiologically trivial: BMD fell to a level similar to that reported after prolonged nonweight bearing or ovariectomy (Kim et al. 2013; Spatz et al. 2013) while the losses in muscle-specific force accounted for approximately one-third of the loss in total muscle force production. The LLC model described here induces substantial alterations to muscle and bone and may be a useful tool for understanding the musculoskeletal deterioration that occurs in human cancer patients.

## References

[b1] Acharyya S, Ladner KJ, Nelsen LL, Damrauer J, Reiser PJ, Swoap S (2004). Cancer cachexia is regulated by selective targeting of skeletal muscle gene products. J. Clin. Invest.

[b2] Acharyya S, Butchbach ME, Sahenk Z, Wang H, Saji M, Carathers M (2005). Dystrophin glycoprotein complex dysfunction: a regulatory link between muscular dystrophy and cancer cachexia. Cancer Cell.

[b3] Agbulut O, Noirez P, Beaumont F, Butler-Browne G (2003). Myosin heavy chain isoforms in postnatal muscle development of mice. Biol. Cell.

[b4] Andrade FH, Reid MB, Allen DG, Westerblad H (1998). Effect of hydrogen peroxide and dithiothreitol on contractile function of single skeletal muscle fibres from the mouse. J. Physiol.

[b5] Argiles JM, Figueras M, Ametller E, Fuster G, Olivan M, de Oliveira CC (2008). Effects of CRF2R agonist on tumor growth and cachexia in mice implanted with Lewis lung carcinoma cells. Muscle Nerve.

[b6] Aulino P, Berardi E, Cardillo VM, Rizzuto E, Perniconi B, Ramina C (2010). Molecular, cellular and physiological characterization of the cancer cachexia-inducing C26 colon carcinoma in mouse. BMC Cancer.

[b7] Azouz SM, Walpole J, Amirifeli S, Taylor KN, Grinstaff MW, Colson YL (2008). Prevention of local tumor growth with paclitaxel-loaded microspheres. J. Thorac. Cardiovasc. Surg.

[b8] Baltgalvis KA, Berger FG, Pena MM, Davis JM, Carson JA (2008). Effect of exercise on biological pathways in ApcMin/+ mouse intestinal polyps. J. Appl. Physiol.

[b9] Banduseela V, Ochala J, Lamberg K, Kalimo H, Larsson L (2007). Muscle paralysis and myosin loss in a patient with cancer cachexia. Acta Myol.

[b10] Benny Klimek ME, Aydogdu T, Link MJ, Pons M, Koniaris LG, Zimmers TA (2010). Acute inhibition of myostatin-family proteins preserves skeletal muscle in mouse models of cancer cachexia. Biochem. Biophys. Res. Commun.

[b11] Brooks SV, Faulkner JA (1988). Contractile properties of skeletal muscles from young, adult and aged mice. J. Physiol.

[b12] Buck M, Chojkier M (1996). Muscle wasting and dedifferentiation induced by oxidative stress in a murine model of cachexia is prevented by inhibitors of nitric oxide synthesis and antioxidants. EMBO J.

[b13] Busquets S, Figueras MT, Fuster G, Almendro V, Moore-Carrasco R, Ametller E (2004). Anticachectic effects of formoterol: a drug for potential treatment of muscle wasting. Cancer Res.

[b14] Cai D, Frantz JD, Tawa NE, Melendez PA, Oh BC, Lidov HG (2004). IKKbeta/NF-kappaB activation causes severe muscle wasting in mice. Cell.

[b15] Diffee GM, Kalfas K, Al-Majid S, McCarthy DO (2002). Altered expression of skeletal muscle myosin isoforms in cancer cachexia. Am. J. Physiol. Cell Physiol.

[b16] Faulkner JA, Brooks SV, Dennis RG (1999). Measurement of recovery of function following whole muscle transfer, myoblast transfer, and gene therapy. Methods Mol. Med.

[b17] Fearon KC (1992). The mechanisms and treatment of weight loss in cancer. Proc. Nutr. Soc.

[b18] Gorselink M, Vaessen SF, Leenders LG, van der Flier I, Kegler D, Caldenhoven E (2006). Mass-dependent decline of skeletal muscle function in cancer cachexia. Muscle Nerve.

[b19] Hardin BJ, Campbell KS, Smith JD, Arbogast S, Smith J, Moylan JS (2008). TNF-alpha acts via TNFR1 and muscle-derived oxidants to depress myofibrillar force in murine skeletal muscle. J. Appl. Physiol.

[b20] Inagaki J, Rodriguez V, Bodey GP (1974). Proceedings: causes of death in cancer patients. Cancer.

[b21] Kandarian S (2008). The molecular basis of skeletal muscle atrophy–parallels with osteoporotic signaling. J. Musculoskelet. Neuronal Interact.

[b22] Kim HY, Alarcon C, Pourteymour S, Wergedal JE, Mohan S (2013). Disruption of claudin-18 diminishes ovariectomy-induced bone loss in mice. Am. J. Physiol. Endocrinol. Metab.

[b23] Llovera M, Garcia-Martinez C, Lopez-Soriano J, Agell N, Lopez-Soriano FJ, Garcia I (1998a). Protein turnover in skeletal muscle of tumour-bearing transgenic mice overexpressing the soluble TNF receptor-1. Cancer Lett.

[b24] Llovera M, Garcia-Martinez C, Lopez-Soriano J, Carbo N, Agell N, Lopez-Soriano FJ (1998b). Role of TNF receptor 1 in protein turnover during cancer cachexia using gene knockout mice. Mol. Cell. Endocrinol.

[b25] Mayo JG (1972). Biologic characterization of the subcutaneously implanted Lewis lung tumor. Cancer Chemother. Rep.

[b26] Mendell JR, Engel WK (1971). The fine structure of type II muscle fiber atrophy. Neurology.

[b27] Mendez J, Keys A (1960). Density and composition of mammalian muscle. Metabolism.

[b28] Murphy KT, Chee A, Gleeson BG, Naim T, Swiderski K, Koopman R (2011). Antibody-directed myostatin inhibition enhances muscle mass and function in tumor-bearing mice. Am. J. Physiol. Regul. Integr. Comp. Physiol.

[b29] Nicholson JR, Kohler G, Schaerer F, Senn C, Weyermann P, Hofbauer KG (2006). Peripheral administration of a melanocortin 4-receptor inverse agonist prevents loss of lean body mass in tumor-bearing mice. J. Pharmacol. Exp. Ther.

[b30] Petrof BJ, Shrager JB, Stedman HH, Kelly AM, Sweeney HL (1993). Dystrophin protects the sarcolemma from stresses developed during muscle contraction. Proc. Natl. Acad. Sci. USA.

[b31] Prochniewicz E, Lowe DA, Spakowicz DJ, Higgins L, O'conor K, Thompson LV (2008). Functional, structural, and chemical changes in myosin associated with hydrogen peroxide treatment of skeletal muscle fibers. Am. J. Physiol. Cell Physiol.

[b32] Reid MB, Lannergren J, Westerblad H (2002). Respiratory and limb muscle weakness induced by tumor necrosis factor-alpha: involvement of muscle myofilaments. Am. J. Respir. Crit. Care Med.

[b33] Rybakova IN, Patel JR, Ervasti JM (2000). The dystrophin complex forms a mechanically strong link between the sarcolemma and costameric actin. J. Cell Biol.

[b34] Spatz JM, Ellman R, Cloutier AM, Louis L, Suva M, van Vliet LJ (2013). Sclerostin antibody inhibits skeletal deterioration due to reduced mechanical loading. J. Bone Miner. Res.

[b35] Stevens ED, Faulkner JA (2000). The capacity of mdx mouse diaphragm muscle to do oscillatory work. J. Physiol.

[b36] Taylor JA, Kandarian SC (1994). Advantage of normalizing force production to myofibrillar protein in skeletal muscle cross-sectional area. J. Appl. Physiol. (1985).

[b37] Tisdale MJ (2009). Mechanisms of cancer cachexia. Physiol. Rev.

[b38] Widrick JJ, Jiang S, Choi SJ, Knuth ST, Morcos PA (2011). An octaguanidine-morpholino oligo conjugate improves muscle function of mdx mice. Muscle Nerve.

[b39] Wilcox PG, Wakai Y, Walley KR, Cooper DJ, Road J (1994). Tumor necrosis factor alpha decreases in vivo diaphragm contractility in dogs. Am. J. Respir. Crit. Care Med.

[b40] Wyke SM, Russell ST, Tisdale MJ (2004). Induction of proteasome expression in skeletal muscle is attenuated by inhibitors of NF-kappaB activation. Br. J. Cancer.

